# Unraveling Potential‐Dependent Mechanistic Pathways of Urea Oxidation on Pt, Ni, and PtNi Electrocatalysts

**DOI:** 10.1002/smll.74274

**Published:** 2026-06-21

**Authors:** Rodrigo G. de Araujo, Diego G. Gigliotti, Geraldo N. Tessaro, Seiti I. Venturini, Joelma Perez

**Affiliations:** ^1^ São Carlos Institute of Chemistry University of São Paulo São Paulo Brazil

**Keywords:** electrocatalysis, nickel, platinum, reaction mechanism, urea oxidation

## Abstract

Elucidating the reaction mechanisms of the urea oxidation reaction (UOR) is key to overcoming kinetic limitations and controlling product selectivity in alkaline media. Here, we combine online electrochemical mass spectrometry and ion chromatography to investigate high‐surface‐area Pt, Ni, and PtNi catalysts supported on carbon. This approach enables the unequivocal identification of seven gaseous products, six derived from UOR (N_2_, NO, N_2_O, CO_2_, HOCN, and NO_2_) and O_2_ from the competing oxygen evolution reaction, as well as three solution‐phase species (NO_2_^−^, NO_3_^−^, and OCN^−^). At low potentials, Pt/C and PtNi/C are catalytically active, with Pt selectively promoting N_2_ production. At higher potentials, NiOOH species dictate the pathways, promoting C─N bond cleavage and forming OCN^−^ and NO as key intermediates in the formation of more oxidized nitrogen products, including NO_2_^−^ as the dominant product for Ni/C and PtNi/C. Together, these findings support a newly proposed mechanistic framework that is potential‐dependent and advances the current understanding of UOR. Remarkably, the PtNi/C catalyst exhibits an early onset of oxidation at low potential while maintaining enhanced catalytic performance at higher potentials, thereby guiding the design of advanced anodes for urea‐assisted water splitting and direct urea fuel cells.

## Graphical abstract

The UOR mechanism on Pt, Ni, and PtNi surfaces shows that at low potentials, only Pt/C and PtNi/C are active, promoting the formation of N_2_ as the dominant product. At higher potentials, C–N bond cleavage becomes predominant, and NO intermediates drive the formation of NO_2_^−^ as the main product on PtNi/C and Ni/C. These findings reveal the synergistic interaction between Pt and Ni, extending the activity window and enhancing UOR performance.

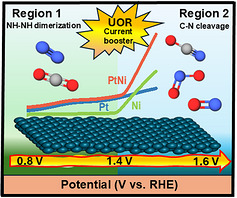

## 1 Introduction

Urea oxidation reaction (UOR) has emerged as a promising route for producing green hydrogen, as urea is an abundant, low‐cost, and nonflammable fuel that can be obtained from industrial and urinary effluents [1]. In this context, UOR has generated growing interest in water‐splitting systems, where its replacement of the oxygen evolution reaction (OER) significantly reduces the overall energy consumption of the process [1, 2, 3]. In addition, the reaction has considerable potential for application in direct urea fuel cells (DUFCs), enabling the electrochemical conversion of urea into electricity and expanding its relevance in environmental mitigation strategies and the valorization of waste streams.

In alkaline media, UOR has been extensively studied on nickel‐based catalysts, and it is widely accepted that the reaction is mediated by NiOOH species, which act as active centers for urea oxidation [3, 4]. However, on pure Ni surfaces, the slow kinetics of urea activation and the high anodic overpotential still pose significant challenges to reaction efficiency. To overcome these challenges, several strategies have been proposed, including the electronic and structural modification of Ni through the incorporation of secondary metals, such as Pt [5, 6, 7], Co [8, 9], or Mo [10, 11], as well as the use of high‐surface‐area conductive supports, with the aim of reducing the onset potential for the reaction and increasing the achievable current density [3].

The incorporation of small amounts of Pt into Ni‐based catalysts has been widely reported to improve UOR performance in alkaline media. For example, Barbosa et al. observed similar onset potentials (∼1.3 V) for Ni/C and Pt‐modified Ni catalysts but significantly higher activity and stability for the optimized bimetallic composition [12]. Likewise, Zhong et al. and Lu et al. reported enhanced performance for Pt─Ni‐based structures compared with their monometallic counterparts, attributing the improvement to synergistic effects between Pt and Ni phases, which may facilitate urea adsorption and activation [6, 7]. More recently, Shi et al. demonstrated that PtNi‐based composite catalysts can reach higher current densities than isolated Ni or Pt materials [13].

These results indicate that modifying Ni‐based catalysts with Pt is an efficient strategy to enhance electrocatalytic activity and stability in UOR, reinforcing the potential of these bimetallic materials for applications in alkaline energy devices. However, the overall process efficiency strongly depends on a detailed understanding of the UOR mechanism and its relationship to catalyst composition, electrolyte composition, applied potential, and operating conditions.

To date, UOR mechanisms have predominantly been proposed for Ni surfaces, where the reaction is described as a highly complex process that mainly proceeds through two routes, N─N coupling and C─N cleavage, each involving multiple elementary steps, as supported by density functional theory (DFT) calculations and in situ spectroscopic and electrochemical techniques [14, 15, 16, 17, 18, 19, 20, 21]. Nevertheless, despite these advances, the reaction pathways underlying UOR remain not yet fully elucidated.

In this context, the use of coupled techniques, such as mass spectrometry (MS) and ion chromatography (IC), is fundamental for identifying gaseous products and soluble species formed during UOR, thereby clarifying the predominant reaction pathways on different catalytic surfaces. Table 1 summarizes the main results reported for Ni‐ and Pt‐based catalysts, including the detection of gaseous and solution‐phase products [14, 15, 16, 21, 22, 23, 24, 25, 26, 27, 28, 29, 30]. Regarding Pt‐modified Ni catalysts, however, the literature has largely concentrated on benchmarking electrocatalytic activity and durability, with comparatively few studies devoted to in‐depth mechanistic analysis or the systematic identification of reaction intermediates and products.

**TABLE 1:** Studies for UOR in Ni and Pt‐based materials, including the detection of reaction products and identification techniques employed: differential electrochemical mass spectrometry (DEMS), online electrochemical mass spectrometry (OLEMS), gas chromatography (GC), and ion chromatography (IC).

Reagent [c / mol L^−1^]	Catalyst	Products	Technique	Refs.
0.5 Urea 1.0 KOH	β‐Ni(OH)_2_	N_2_ and ^a^N_2_	DEMS	[14]
0.33 Urea 1.0 KOH	I‐NiS@NF	N_2_, O_2_, and CO_2_	DEMS	[22]
0.33 Urea 1.0 KOH	Ni(OH)_2_	N_2_ and ^a^N_2_	DEMS	[15]
0.5 Urea 1.0 KOH	Ni‐TPA@NiSe/NF	N_2_ and ^a^N_2_	DEMS	[16]
0.33 Urea 1.0 KOH	Ni‐SO_x_	N_2_ and O_2_	DEMS	[26]
0.33 Urea 1.0 KOH	Ni_1_@NC	N_2_, NCO^−^, and NO_2_^−^	DEMS/IC	[21]
0.33 Urea 1.0 KOH	NiCoGe	N_2_, O_2_, NO_2_^−^, CNO^−^, NO_3_^−^, and CO_3_^2−^	DEMS/IC	[23]
0.33 Urea 1.0 KOH	Ni foam	CO_3_^2−^, NO_3_^−^, NO_2_^−^, N_2_, ^a^N_2_, and ^a^N_2_O	GC‐MS/IC	[29]
0.33 Urea 1.0 KOH	Ni(OH)_2_/NF	NCO^−^, NO_2_^−^, and NO_3_^−^	IC	[28]
0.1 Urea 0.1 HClO_4_	Pt	N_2_ and CO_2_	DEMS	[24]
0.03 Urea phosphate buffer	Pt	^15^NO, ^15^N_2_O, ^15^N_2_, and ^15^NO_2_	DEMS	[27]
1.0 Urea 3.0 KOH	PtPdNi/C	NO, CO_2_/N_2_O, and NH_3_	DEMS	[30]
1.0 Urea 1.0 NaOH	Pt/C, Ni/C, and PtNi/C	O_2_, N_2_, NO, N_2_O, NO_2_, ^b^HOCN, ^b^CO_2_, OCN^−^, NO_2_^−^, and NO_3_^−^	OLEMS/IC	This Work

^a)^ ^14^N^15^N and ^15^N_2_ labeled urea

^b)^ in acidic medium

In light of these gaps, the present work aims to elucidate the reaction mechanisms of UOR on Pt, Ni, and PtNi surfaces. Instead of focusing primarily on catalyst novelty, this study employs well‐defined commercial Pt, Ni, and PtNi catalysts supported on carbon to establish fundamental structure–property–function relationships and to provide mechanistic insights that can guide the rational design of advanced electrocatalysts for low‐potential UOR applications. To this end, cyclic voltammetry and chronoamperometry are employed, both coupled with OLEMSand IC, which are used to detect gaseous and soluble species, respectively. The comparison of the results with previously reported data (see Table 1) highlights the advances this study provides toward the mechanistic understanding of the reaction. For the first time, the combination of OLEMS and IC techniques enables, in a systematic manner, the simultaneous identification of multiple products formed during UOR, depending on the electrode material and the applied potential. This approach allows the individual roles of Pt/C, Ni/C, and the bimetallic PtNi/C system to be established in the different reaction pathways involved in UOR, revealing how catalyst composition influences activity, selectivity, and reaction pathways.

## 2 Results and Discussion

**FIGURE 1:**
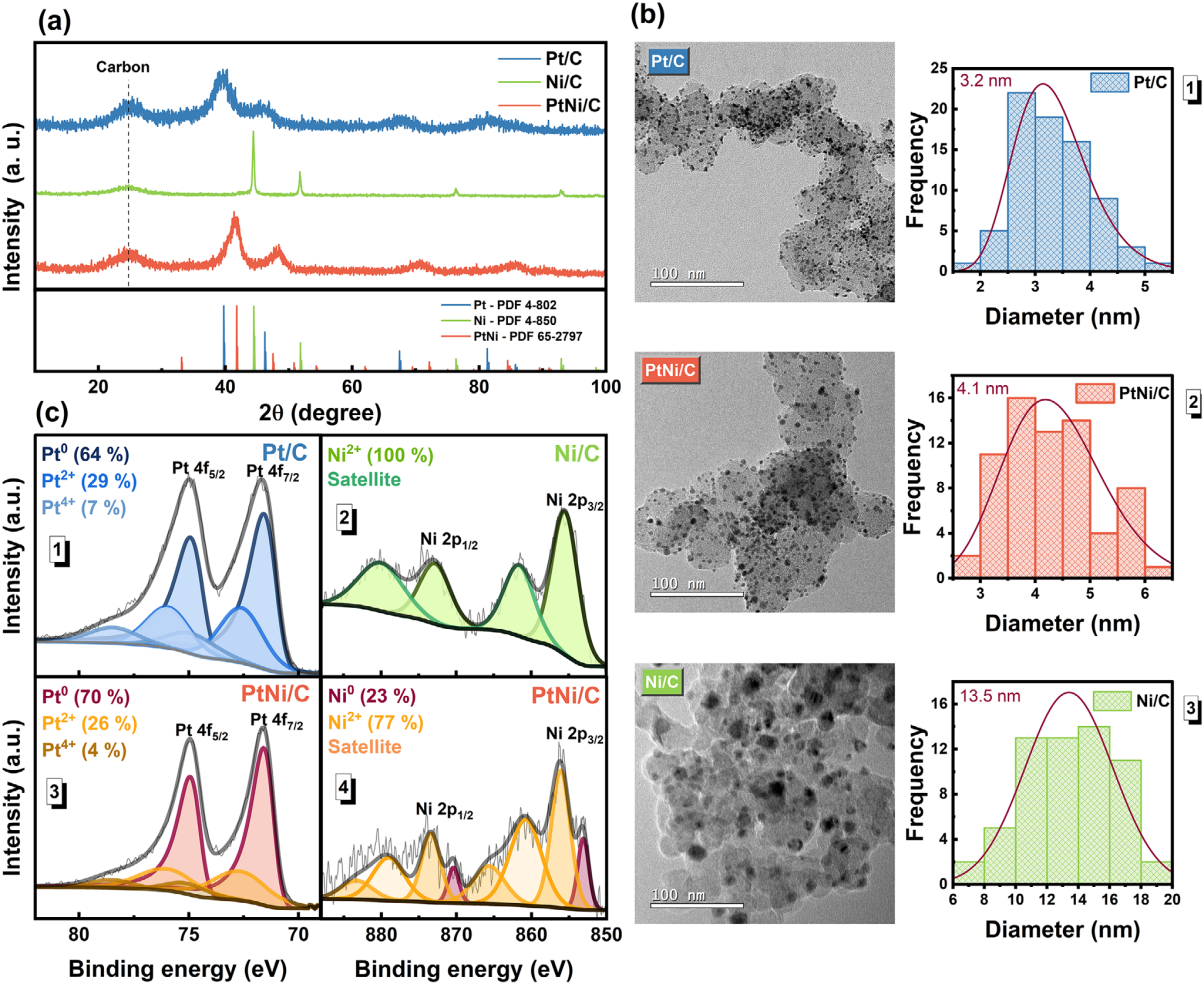
Characterization of the Pt/C, Ni/C, and PtNi/C electrocatalysts: (a) XRD results and patterns; (b) TEM images; and (c) XPS spectra.

Figure 1a presents the X‐ray diffractograms of the Pt/C, Ni/C, and PtNi/C catalysts, along with the reference diffraction patterns from the International Centre for Diffraction Data (ICDD) Powder Diffraction File (PDF) database. The XRD patterns confirmed the FCC structure of Pt in Pt/C [31, 32]. Similarly, the Ni/C catalyst showed reflections attributed to metallic nickel in the presence of FCC‐structured oxides [32]. The formation of a Pt─Ni alloy in PtNi/C was evidenced by the shift of the Pt diffraction peaks toward higher angles [33]. Thermogravimetric analysis (TGA) and energy dispersive X‐ray spectroscopy (EDX) analyses confirmed metallic loadings close to 20 wt.% for all catalysts (Figure S1 and Table S1), with PtNi/C exhibiting a total metal content of approximately 18.7 wt.% and a Pt: Ni atomic ratio of approximately 60:40. Transmission electron microscopy (TEM) analysis (Figure 1b1–3) showed well‐dispersed, predominantly spherical nanoparticles on carbon, with average particle sizes of 3.2 nm (Pt/C), 4.1 nm (PtNi/C), and 13.5 nm (Ni/C). X‐ray Photoelectron Spectroscopy (XPS) analysis (Figure 1c1‐4) revealed Pt^0^/Pt^2^^+^/Pt^4^^+^ species in Pt/C [34], predominantly Ni^2^^+^ in Ni/C [35], and the coexistence of Pt^0^ and Ni^0^ in PtNi/C with an increased metallic Pt fraction (Table S2), thereby supporting PtNi alloy formation in agreement with the XRD results.

**FIGURE 2:**
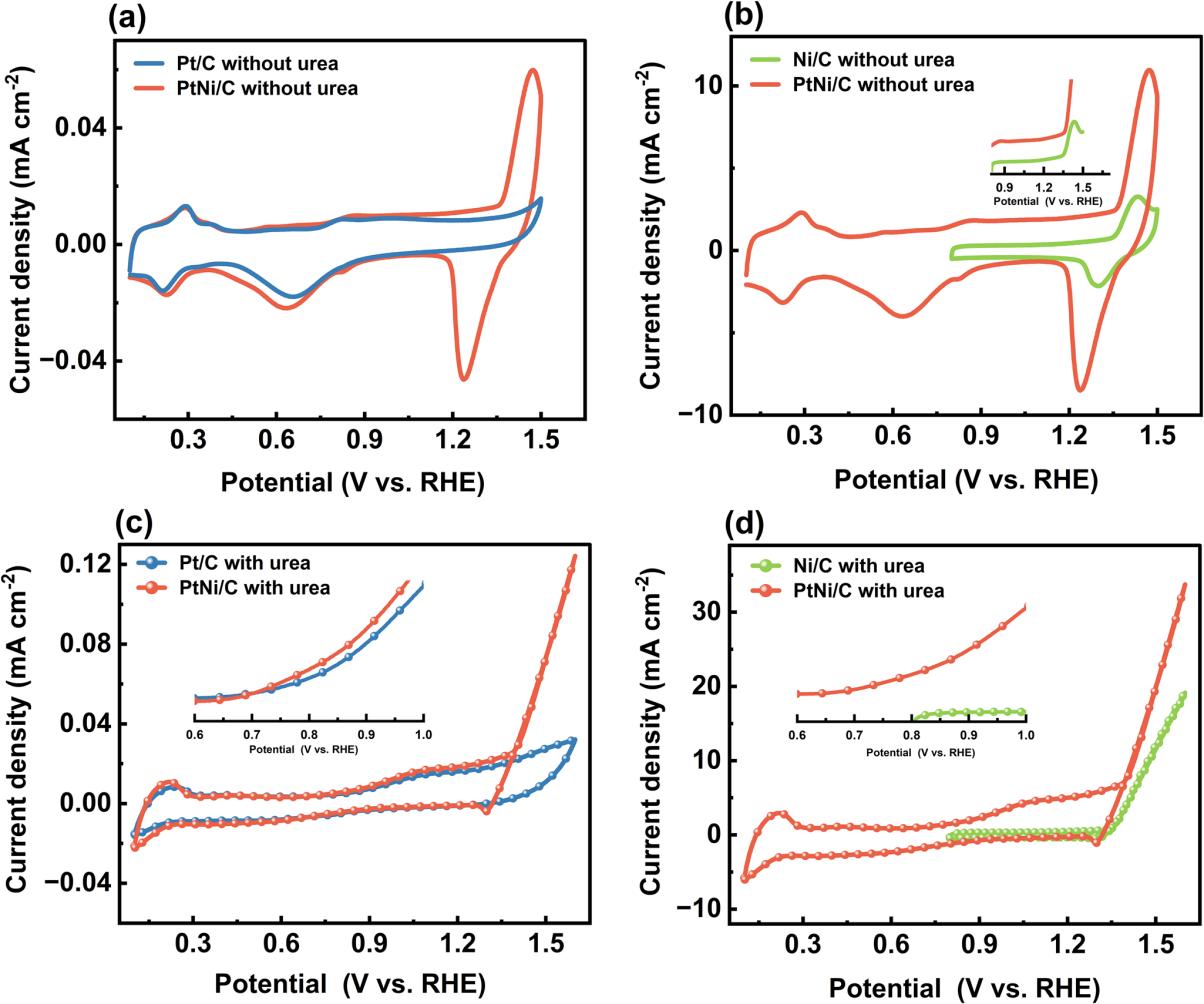
Cyclic voltammograms of the electrocatalysts recorded at a scan rate of 5 mV s^−^^1^: (a) in 1.0 mol L^−^^1^ NaOH for Pt/C and PtNi/C electrocatalysts; (b) in 1.0 mol L^−^^1^ NaOH for Ni/C and PtNi/C electrocatalysts; (c) in 1.0 mol L^−^^1^ NaOH + 1.0 mol L^−^^1^ urea for Pt/C and PtNi/C, normalized by electrochemical surface active area; and (d) in 1.0 mol L^−^^1^ NaOH + 1.0 mol L^−^^1^ urea for Ni/C and PtNi/C, normalized by geometric area.

Figure 2a,c shows the results of cyclic voltammetry (CV) performed in 1.0 mol L^−1^ NaOH for the Pt/C and PtNi/C catalysts in the absence and presence of urea, respectively, where normalization by ECSA was adopted because the Pt sites dominate the catalytic response in the low potential region. In Figure 2a, the Pt/C and PtNi/C catalysts exhibited the characteristic peaks of Pt hydrogen adsorption/desorption in the potential region of 0.1–0.35 V, followed by the formation of platinum oxides from approximately 0.7 V and the onset of OER for the Pt catalyst above 1.4 V [36]. On the other hand, for the PtNi/C catalyst, an oxidation peak at approximately 1.4 V was observed, which was attributed to the conversion of Ni(OH)_2_ to NiOOH, while the reduction from Ni^3+^ to Ni^2+^ was observed at approximately 1.25 V [3, 4, 37].

The CV results obtained in the absence and presence of urea for the Ni catalyst, compared with PtNi/C, are shown in Figure 2b,d, respectively, with current normalized by geometric area. In Figure 2b, the oxidation and reduction potentials of the Ni(OH)_2_/NiOOH redox pair were observed in the same potential region for Ni/C and PtNi/C. Additionally, the onset potentials for NiOOH formation were consistent with values reported in the literature, including recent Raman spectroscopy studies that indicate a potential of approximately 1.4 V [14, 38, 39]. The results in the absence and presence of urea for the Pt/C, PtNi/C, and Ni/C catalysts, normalized by geometric area, are presented in Figure S2.

In the presence of urea, Figure 2c shows the CV curves for the Pt/C and PtNi/C catalysts, while Figure 2d shows those for the Ni catalyst. The onset potential of UOR was observed at approximately 0.8 V for the Pt/C and PtNi/C catalysts (Figure S2), further supported by MS results. Across the entire potential range investigated, the PtNi/C catalyst exhibited higher activity toward UOR compared with Pt, with a sharp increase in current from 1.4 V, coinciding with the onset of UOR for the Ni/C catalyst (Figure 2d), which was associated with the formation of NiOOH species [3, 4, 37]. The PtNi/C catalyst also exhibited higher activity than Ni/C between 1.4 and 1.6 V, even with a lower nickel content (Table S1). Previous studies evaluating PtNi catalysts for UOR [6, 7, 12, 13] corroborated our results, indicating that Pt‐modified Ni catalysts exhibit superior activity compared with monometallic Ni and Pt catalysts. However, these studies reported reaction onset at 1.30–1.35 V and did not identify activity in the low‐potential region, as observed in the present study.

The backward scan analysis in Figure 2c indicated hysteresis over nearly the entire potential range studied for the Pt catalyst, suggesting progressive deactivation of the Pt surface in the presence of urea. The PtNi/C catalyst showed behavior similar to that of Pt in the low‐potential region (0.8–1.4 V), indicating that the adsorbed species that block the surface also adsorbed strongly on this surface. Conversely, in the range of 1.4–1.60 V, both the Ni/C and PtNi/C catalysts did not show hysteresis between the forward and backward scans (Figure 2d). Backward scan analysis of Ni and Pt materials has not been previously reported in the literature.

To gain deeper insight into the behavior of these surfaces toward the UOR, the OLEMS technique was employed to monitor the mass spectrometric (MS) signals of gaseous products during UOR by cyclic voltammetry (CV‐MS) and chronoamperometry (CA‐MS). The mass signals monitored during CV‐MS and CA‐MS were *m/z* 28, 30, 32, 44, and 46, assigned to species N_2_, NO, O_2_, CO_2_/N_2_O, and NO_2_, respectively, in the presence and absence of 1.0 mol L^−1^ urea. The measurement protocols are summarized in Table S3 and Table S4. It is important to note that the OLEMS data were interpreted in a semi‐quantitative manner, primarily to identify relative trends in gaseous product evolution during UOR. Nevertheless, since all measurements were performed under identical experimental conditions and reproduced in independent experiments, the relative signal ratios between the detected species remained consistent across measurements, providing reliable information regarding comparative changes in gaseous product distribution and selectivity.

**FIGURE 3:**
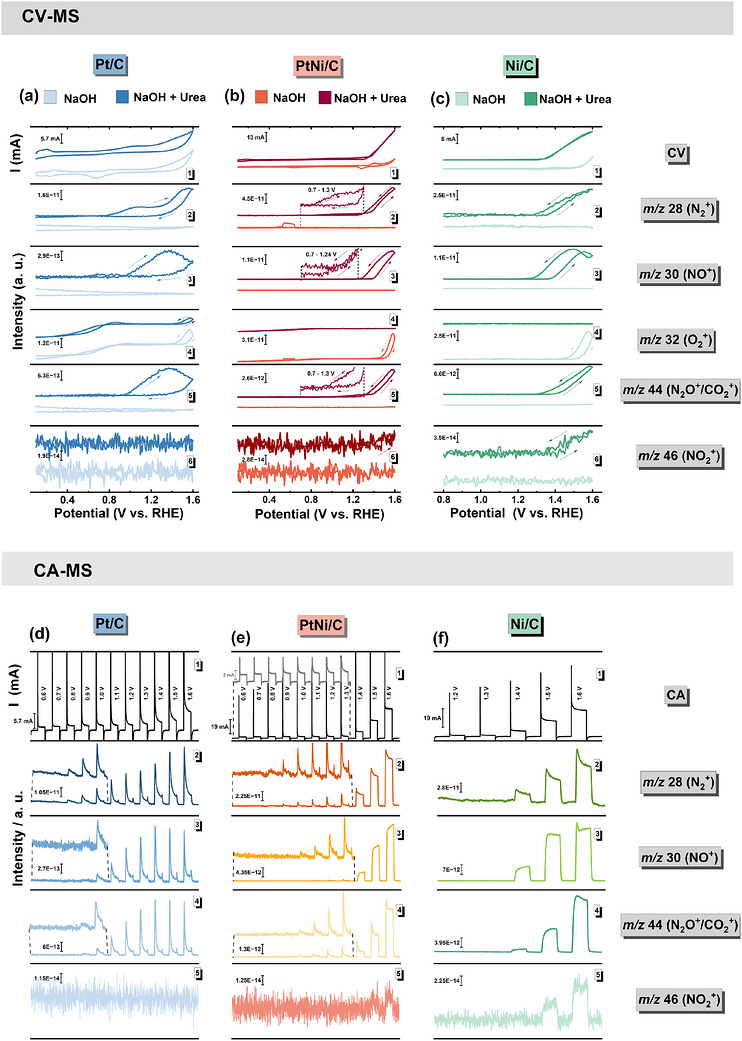
CV‐MS and CA‐MS results for the Pt/C, PtNi/C, and Ni/C. (a–c) CV‐MS results recorded at a scan rate of 1 mV s^−1^ in 1.0 mol L^−1^ NaOH (lower traces) and in 1.0 mol L^−1^ NaOH + 1.0 mol L^−1^ urea (upper traces), showing the *m/z* signals 28, 30, 32, 44, and 46. (d–f) CA‐MS results obtained in 1.0 mol L^−1^ NaOH + 1.0 mol L^−1^ urea, displaying the m/z signals 28, 30, 44, and 46.

The CV‐MS and CA‐MS results for the Pt/C, PtNi/C, and Ni/C catalysts are shown in Figure 3a–f, respectively. At the top of each panel, the corresponding electrochemical CV and CA curves are presented, followed by the associated CV‐MS and CA‐MS responses. For each result, the upper traces correspond to the presence of urea, whereas the lower traces correspond to its absence.

It is worth noting that, in the absence of urea, the CV‐MS results did not indicate the formation of N_2_, NO, N_2_O, and NO_2_ for any of the catalysts evaluated, as expected, confirming that the gaseous species originated exclusively from UOR. This finding underscored the importance of gas and solution purity, as well as the need for rigorous measurement protocols [40].

For nitrogen (*m/z* 28), the CV‐MS results for Pt/C, PtNi/C, and Ni/C are presented in Figure 3a2, 3b2 and 3c2, respectively. For Pt/C and PtNi/C catalysts, the onset potential of N_2_ formation was approximately 0.80 V. On the Pt surface, at around 1.05 V, the N_2_ signal reached a slight plateau, then decreased and, from 1.3 V onward, increased continuously. During the backward scan, a significant reduction in N_2_ production was observed, indicating catalytic deactivation, corroborating the electrochemical results. Similarly, Bolzan et al. observed the onset of urea oxidation and N_2_ formation at approximately 0.75 V, accompanied by progressive surface deactivation during the backward scan [27]. Bezerra et al. detected the evolution of *m/z* 28 starting at approximately 0.8 V and also reported pronounced surface deactivation during the backward scan [24]. To the best of our knowledge, no MS studies have previously reported *m/z* 28 monitoring for PtNi/C or related bimetallic surfaces.

In contrast, on the Ni surface (Figure 3c2), nitrogen detection occurred at 1.4 V, with a continuous increase in the N_2_ signal throughout the entire scan up to 1.6 V, corroborating the electrochemical result. In this potential region, the PtNi/C catalyst exhibited an N_2_ production profile similar to that observed for the Ni surface, with the backward scan reproducing the behavior of the forward scan and no evidence of catalytic deactivation for either material. These findings are consistent with recent studies that reported the onset of the *m/z* 28 signal at approximately 1.4 V during UOR in alkaline media, indicating that N_2_ formation occurred only at relatively high potentials across different Ni‐based surfaces [23, 26]. Together, these reports supported our observation that Ni‐based materials did not exhibit significant catalytic deactivation under these conditions.

The CA‐MS results (Figure 3d2, 3e2 and 3f2) for the Pt/C, PtNi/C, and Ni/C catalysts, respectively, confirmed the CV‐MS onset observations. At all applied potentials, the CA‐MS for the Pt electrocatalysts showed that N_2_ production initially increased and then decreased over the polarization time. For the PtNi/C catalyst, the N_2_ production profile was similar to that observed for Pt/C in the potential range of 0.8–1.4 V, indicating surface deactivation. Between 1.4 and 1.6 V, however, the N_2_ production began to resemble that of the Ni catalyst, where the MS results indicated slight deactivation in N_2_ production over time. To date, no reports have described chronoamperometry coupled with mass spectrometry measurements for Pt and PtNi surfaces.

Previous DEMS studies on Ni‐based catalysts have confirmed N_2_ formation during UOR, often using ^15^N‐labeled urea to distinguish inter‐ and intramolecular N─N coupling pathways, but without a systematic potential‐resolved analysis. In general, N_2_ evolution was reported at relatively high potentials (∼1.45–1.6 V), sometimes accompanied by slight deactivation over time. Some studies also reported high selectivity toward UOR, whereas others confirmed N_2_ generation through isotopic distribution, albeit without detailed electrochemical protocols or precise potential control [14, 16, 21, 22].

Nitric oxide (*m/z* 30) CV‐MS results for Pt/C, PtNi/C, and Ni/C are presented in Figure 3a3, 3b3 and 3c3, respectively. During the forward scan, the onset of NO formation was observed at approximately 1.0 V on Pt and 1.1 V on PtNi/C. For Pt, NO production increased to a maximum at around 1.3 V, then decreased up to 1.6 V, and surface deactivation was observed during the backward scan. For PtNi/C, NO production increased continuously up to 1.6 V. On the Ni surface, NO was detected from 1.4 V, reaching a maximum at approximately 1.55 V and showing a slight reduction up to 1.6 V. In contrast, the PtNi/C catalyst showed increased NO production during the backward scan up to approximately 1.3 V, a trend also observed for the Ni surface. The literature reports only one study identifying NO formation during UOR on a PtPdNi/C catalyst [30], in which NO production was observed exclusively at high potentials. In contrast, the results demonstrated, for the first time, the formation and potential‐dependent evolution of NO on Pt, PtNi, and Ni surfaces, including detection at lower potentials and distinct behaviors between forward and backward scans.

The CA‐MS results for *m/z* 30 (Figure 3d3, 3e3 and 3f3) confirmed the onset potential obtained by CV‐MS. At all applied potentials for Pt/C, the NO production peak was followed by a decrease over the polarization time. For the PtNi/C catalyst, the NO formation profile was similar to that observed for Pt between 1.0 and 1.3 V. However, in the range of 1.4–1.6 V, the catalyst behavior resembled that of the Ni surface, characterized by a significant increase in NO production compared to Pt.

The *m/z* 44 signal could arise from either N_2_O or CO_2_, both possible UOR products. In DEMS studies, this contribution is generally regarded as ambiguous; however, in alkaline media, CO_2_ is expected to be mostly converted to carbonate (CO_3_^2−^) [28, 30]. Some studies detected CO_3_^2−^ by IC [23, 29], while others identified it by NMR [28], all reporting only small amounts and none including this species in the overall faradaic efficiency calculation. Complementary CA‐MS experiments performed in acidic and alkaline electrolytes (Figure S3) confirmed that CO_2_ formation occurred only under acidic conditions.

Nitrous oxide (*m/z* 44) CV‐MS results for Pt/C, PtNi/C, and Ni/C are presented in Figure 3a5, 3b5 and 3c5. The onset of detection occurred around 1.0 V for the Pt and PtNi surfaces. For Pt, the maximum production occurred near 1.4 V, followed by a decrease in intensity, and surface deactivation was observed during the backward scan. For PtNi/C, N_2_O formation increased continuously with potential, but no significant deactivation was observed during the backward scan until 1.2 V. On the Ni surface, detection began at approximately 1.4 V, and the production of this species continued to increase across the applied potential, with a slight increase during the backward scan. Bolzan et al. demonstrated that, for Pt, N_2_O formation began around 0.8 V [27]. Li et al. employed GC‐MS to identify traces of N_2_O on Ni foam surfaces [29]. Other authors, although working in an alkaline medium, attributed *m/z* 44 predominantly to CO_2_ in Ni‐based catalysts without discussing the possible contribution of N_2_O [15, 22].

The CA‐MS measurements for the *m/z* 44 signal (Figure 3d4, 3e4 and 3f4) confirmed the onset potentials observed by CV‐MS. Pt exhibited an initial rise in N_2_O production followed by a gradual decay. For the PtNi/C catalyst, the N_2_O formation profile was similar to that of Pt between 1.0 and 1.3 V. However, in the 1.4–1.6 V range, its behavior progressively shifted toward that of Ni, with a pronounced increase in N_2_O production.

Nitrogen dioxide (*m/z* 46) CV‐MS results for the Pt/C, PtNi/C, and Ni/C catalysts are presented in Figure 3a6, 3b6 and 3c6, respectively. No formation was observed on the Pt surface during the forward and backward scans. For the PtNi/C and Ni/C catalysts, NO_2_ was detected at low intensity from approximately 1.5 V, with the Ni/C catalyst showing slightly higher production. During the backward scan, the Ni surface maintained its activity, without any observable decline in NO_2_ production. In the literature, the *m/z* 46 signal during UOR was reported only on Pt in phosphate buffer at high potentials, with a weak signal [27]. The present results indicated slight NO_2_ production on PtNi/C and more pronounced formation on Ni/C, suggesting that the presence of Ni promoted NO_2_ formation.

The CA‐MS results for the *m/z* 46 signal (Figure 3d5) confirmed that this species did not form on Pt/C at any of the investigated potentials. In contrast, low‐intensity *m/z* 46 signals were detected for PtNi/C and Ni/C catalysts at 1.5 and 1.6 V (Figure 3e5 and 3f5).

The competition between UOR and OER in alkaline media was a limiting factor for process efficiency. The formation of the oxygen *m/z* 32 signal was evaluated by CV‐MS for Pt/C, PtNi/C, and Ni/C in the presence (upper panels) and absence (lower panels) of urea (Figure 3a4, 3b4 and 3c4).

In the absence of urea, all catalysts showed OER onset potentials in the range of 1.5–1.6 V. For Pt‐based catalysts, OER began after the formation of platinum surface oxides [41]. On Ni‐containing surfaces, the reaction proceeded via nickel oxyhydroxide formation, recognized as the active centers for OER [42]. In the presence of urea, the Pt/C catalyst exhibited a decrease in O_2_ production. No *m/z* 32 signal was detected on PtNi or Ni surfaces, indicating high selectivity toward UOR and effective OER suppression within the studied potential range, consistent with previous reports on nickel‐based catalysts [22].

**FIGURE 4:**
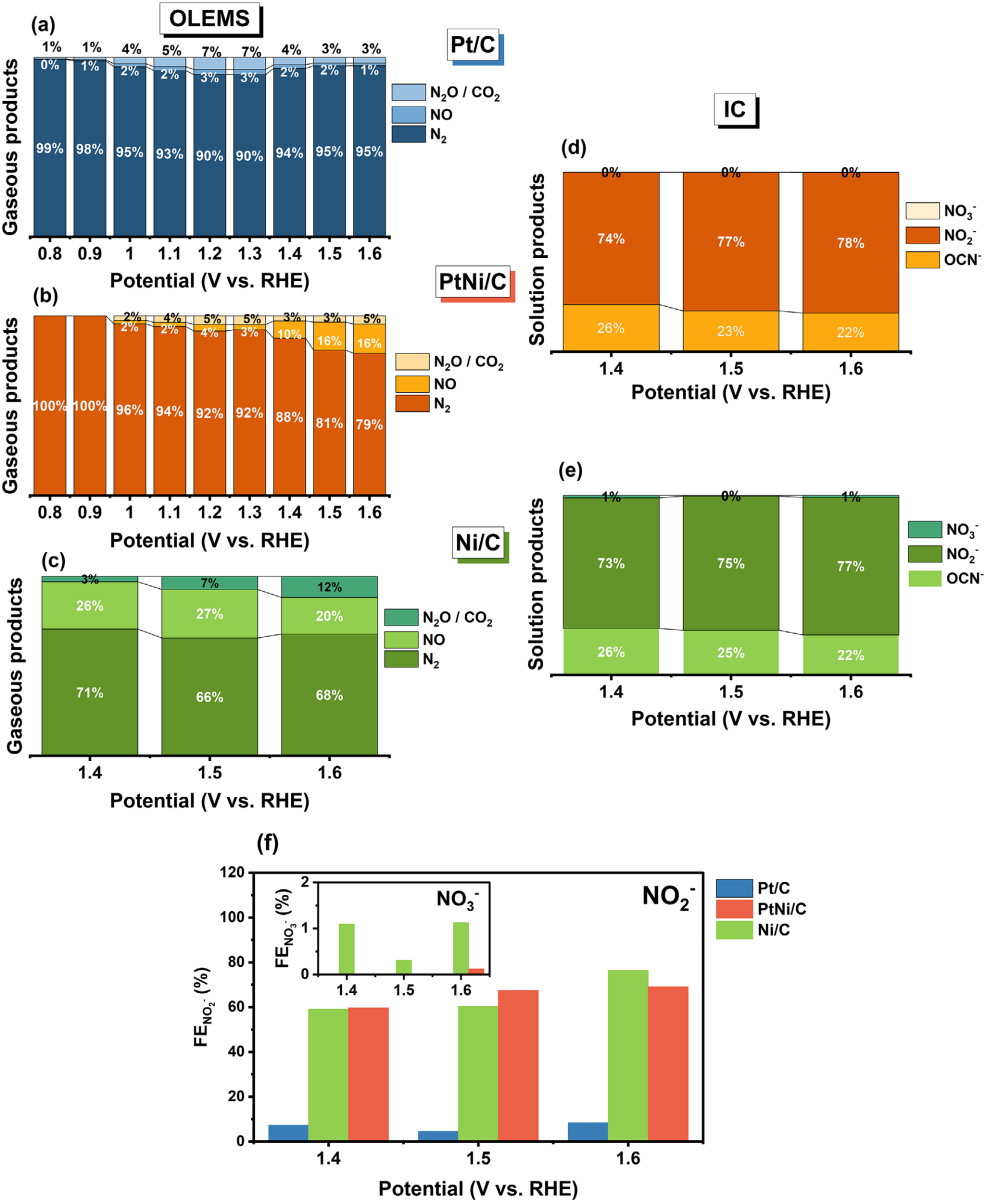
(a–c) Comparison of the gaseous product distribution detected by OLEMS for the electrocatalysts: (a) Pt/C, (b) PtNi/C, and (c) Ni/C. (d–e) Distribution of solution products (NO_2_^−^, OCN^−^, and NO_3_^−^) determined by IC for (d) PtNi/C and (e) Ni/C. (f) Faradaic efficiency for the NO_2_^−^ and NO_3_^−^ anions.

Figure 4a–c presents the potential‐dependent selectivity of gaseous UOR products, showing the relative distribution of N_2_, NO, and N_2_O detected by OLEMS. For Pt/C and PtNi/C catalysts, N_2_ predominated across the entire potential range, whereas NO and N_2_O remained minority species; NO_2_ was negligible and therefore excluded from comparative analysis. Above 1.4 V, Ni activity became evident, characterized by a lower relative fraction of N_2_ and increased formation of NO and N_2_O. These differences reflected catalyst‐dependent reaction pathways and highlighted the importance of correlating gaseous and soluble products to elucidate the UOR mechanism. Although current densities were low in this potential region, the predominance of N_2_ suggests high selectivity toward nitrogen formation. Therefore, IC analyses were performed to identify and quantify soluble nitrogen‐containing products formed during UOR.

IC analyses were performed for Pt/C, PtNi/C, and Ni/C catalysts using CA at 1.1, 1.2, 1.3, 1.4, 1.5, and 1.6 V (3600 s at each potential) in 1.0 mol L^−1^ urea, enabling identification and quantification of the anions OCN^−^, NO_2_^−^, and NO_3_^−^. Calibration curves are shown in Figure S4a–c, and the amounts of anionic products as a function of the potential for the Pt/C, PtNi/C, and Ni/C catalysts are shown in Figure S5a‐c.

For Pt/C, only the NO_2_^−^ and OCN^−^ ions were detected, in significantly lower quantities than for PtNi/C and Ni/C catalysts; to our knowledge, no prior IC measurements for UOR on Pt have been reported. CA‐MS experiments in acidic and alkaline media (Figure S3a,b) confirmed that HOCN formed exclusively under acidic conditions.

The anion ratios produced at 1.4, 1.5, and 1.6 V for PtNi/C and Ni/C are shown in Figure 4d,e. Nitrate was the minority product, corroborating OLEMS results that showed low NO_2_ intensity, indicating that NO_2_ acted as a precursor for the formation of NO_3_^−^ [43]. Wang et al. and Tatarchuk et al. similarly reported only trace amounts of NO_3_^−^ at higher potentials on modified Ni surfaces [23, 28].

The dominant soluble products for PtNi/C and Ni/C were OCN^−^ and NO_2_^−^, detected from 1.3 V (PtNi) and 1.4V (Ni). Literature reports OCN^−^ detection on modified Ni surfaces from 1.4 V [23, 28]. Wang et al. observed high selectivity toward NO_2_^−^ for NiCoGe catalysts at 1.4–1.6 V, achieving Faradaic efficiencies close to 80% [23]. Other Ni‐based catalysts showed similar NO_2_^−^ selectivity, although at higher potentials (∼1.6 V) [21, 28, 29]. The detected OCN^−^ is not a product of direct electrochemical oxidation, but rather a product of C‐N bond cleavage during UOR; therefore, its formation was not included in the faradaic efficiency calculations [23, 28].

Figure 4f presents the Faradaic efficiency (FE) values obtained for NO_2_^−^ and NO_3_^−^ at potentials of 1.4, 1.5, and 1.6 V for the Pt/C, PtNi/C, and Ni/C catalysts. The contribution of FE from NO_3_^−^ was very low for the Ni/C (approximately 1%) and PtNi/C (approximately 0.2%) catalysts and was not detected for Pt (inset in Figure 4e). The FE for NO_2_^−^ ranged from 60–80% for the PtNi/C and Ni/C catalysts, whereas for Pt it remained below 5%. This behavior was consistent with previous studies on Ni‐based catalysts [21, 23, 28, 29].

Based on all the evidence presented thus far, the relevance of these findings became particularly evident when compared with previous reports (Table 1). For Pt/C and PtNi/C, in addition to the scarcity of studies that simultaneously identified both gaseous and soluble species during UOR, few studies have provided detailed mechanistic interpretations of the reaction pathways. On the other hand, for Ni‐based catalysts, multiple reaction pathways have been reported [14, 15, 16, 19, 28, 29].

Herein, the comprehensive analysis established a potential‐dependent relationship between electrocatalyst composition and UOR selectivity, substantially advancing the mechanistic understanding of the reaction in alkaline media on Pt, PtNi, and Ni surfaces. Accordingly, the UOR mechanism was discussed in two electrochemical regions: Region I (0.8–1.4 V) and Region II (1.4–1.6 V), reflecting the distinct surface chemistry and catalytic behavior of each material. The division of these potential regions was based primarily on the onset of Ni activity and was used to highlight changes in the predominant reaction pathways across the investigated potential range.

Thereafter, Figure 6 summarizes the proposed mechanistic framework, highlighting the experimentally identified intermediates and products. The reaction was proposed to begin with urea adsorption, followed by the initial NH_2_ dehydrogenation step.

In Region I, only the Pt/C and PtNi/C catalysts were active, and nitrogen was the predominant product, mainly following the route highlighted in green in Figure 6, which was divided into two possible pathways: (a) intramolecular N─N coupling and (b) dimerization of adjacent NH─NH species. The first pathway, intramolecular N─N coupling, this proposal involves the formation of heterocyclic intermediates (pathways a1 and a2), followed by the formation of N_2_ and CO_2_ (a3). The second pathway, referred to as dimerization of adjacent NH─NH, involves the urea molecule initially adsorbing via two NH_2_ groups on adjacent sites of the catalytic surface (pathways b1 and b2). Subsequently, nucleophilic attack by OH^−^ on the carbonyl was proposed to occur, promoting C─N bond cleavage (b3). This step was followed by dehydrogenation of the C─OH group and a second C─N bond cleavage, resulting in CO_2_ release (b4) and leaving NH species adsorbed on the surface (b5). These NH species were proposed to dimerize to form N_2_H_2_, which was subsequently oxidized to N_2_ (b6).

The intramolecular N─N coupling pathway is one of the mechanisms most proposed in the literature for N_2_ formation during urea oxidation. This pathway has been supported by both DFT studies and experiments using ^15^N‐labeled compounds, which showed that the nitrogen atoms forming N_2_ originate predominantly from a single urea molecule [14, 15, 16, 29]. However, these findings do not exclude the possibility of N_2_ formation through the dimerization of adjacent adsorbed NH─NH species. In this context, the second pathway may be favored by increasing the availability of active sites on the catalyst surface and promoting the adsorption of the second N atom, which makes the N─N coupling step more energetically accessible and consequently suppresses C─N bond cleavage [28, 38]. Additionally, these studies suggest that the coupling of neighboring adsorbed NH_x_ species represents a more chemically consistent pathway, as it avoids the formation of an unstable heterocyclic intermediate [29, 44]. On the other hand, Li et al. [29], combining ^15^N isotope labeling and urea analogs, identified the intramolecular N─N coupling mechanism as the dominant route for nitrogen production compared to the adjacent NH─NH coupling pathway. Therefore, despite significant advances, the distinction between these possible mechanisms for N_2_ formation remains unresolved. To the best of our knowledge, there is still no direct experimental evidence in the literature identifying the key reaction intermediates involved in either pathway.

Furthermore, our results show that only Pt and PtNi catalysts are active in the low‐potential region, while both materials exhibit similar particle sizes. According to the XRD data, Ni incorporation modifies the Pt lattice parameters and modifies the urea adsorption energy, which may alter the availability of adjacent active sites and contribute to the subtle enhancement in catalytic activity observed for PtNi. These findings further support the possibility that adjacent NH─NH coupling may play an important role in the low‐potential UOR pathway.

Moreover, the progressive decay in both current density and product formation during chronoamperometric measurements in Region I (Figure 3d–e) indicates a surface deactivation process. Importantly, the intermittent potential steps at 0.05 V promoted recovery of the catalytic activity before the subsequent polarization step, suggesting that the deactivation process is reversible and associated with the formation and removal of adsorbed species. This behavior could be explained by the accumulation of strongly adsorbed nitrogen species (N_ads_) promoted by successive dehydrogenation steps, as supported by DFT calculations [45]. While N_ads_ formation provided a plausible explanation for the observed current decay, the involvement of other nitrogen‐ or oxygen‐containing intermediates could not be excluded, thereby warranting further investigation.

In Region II, the Pt/C catalyst showed negligible activity, indicating its limitation under more oxidizing conditions, where the reaction remained predominantly on the green pathway and was accompanied by pronounced surface deactivation. In contrast, PtNi/C and Ni/C exhibited a pronounced enhancement in catalytic activity, along with a shift in the preferential reaction mechanism, associated with the significant formation of the soluble species OCN^−^ and NO_2_^−^ (Figure 4d,e), and, to a lesser extent, the gaseous species NO, N_2_O, and NO_2_, as well as NO_3_^−^. These observations indicated the predominance of the pathway highlighted in blue in Figure 6.

In this pathway, referred to as the C─N cleavage route, cleavage of the C─N bond was proposed to result in the formation of OCN^−^ (chemical step pathway c2) and NH_ads_ (pathway c3). The NH_ads_ species were proposed to undergo successive oxidation steps, giving rise to oxygenated nitrogen products, NO, N_2_O, NO_2_, NO_2_^−^, and NO_3_^−^_as identified by OLEMS and IC. Specifically: (i) NO production was proposed to occur via the reaction of NH_ads_ with OH^−^ (pathways c4 and c5); (ii) N_2_O production was proposed to result from the coupling of NH_ads_ with NO in the presence of OH^−^ (pathway c6); (iii) NO_2_ production was attributed to the oxidation of NO_ads_ via reaction with OH^−^ (pathway c8 and c9); (iv) NO_2_^−^ production was proposed to occur through the reaction of NO with OH^−^ (pathway c7) and/or the hydrolysis of NO_2_ (pathway c10); and (v) NO_3_^−^ production was attributed to further hydrolysis of NO_2_ (pathway c10) or oxidation of nitrite (pathway c11). Additionally, in Region II, a minor amount of N_2_ was detected, which was associated with the green pathway.

The possible pathways for the formation of NO_2_^−^ and NO_3_^−^ warranted special consideration. NO_3_^−^ was detected by IC from 1.5 V, simultaneously with the detection of NO_2_ by OLEMS, both at low intensities, which was consistent with its formation via NO_2_ hydrolysis (pathway c10) [46]. However, the significant increase in NO_2_^−^ production from 1.4 V could not be explained solely by this pathway.

**FIGURE 5:**
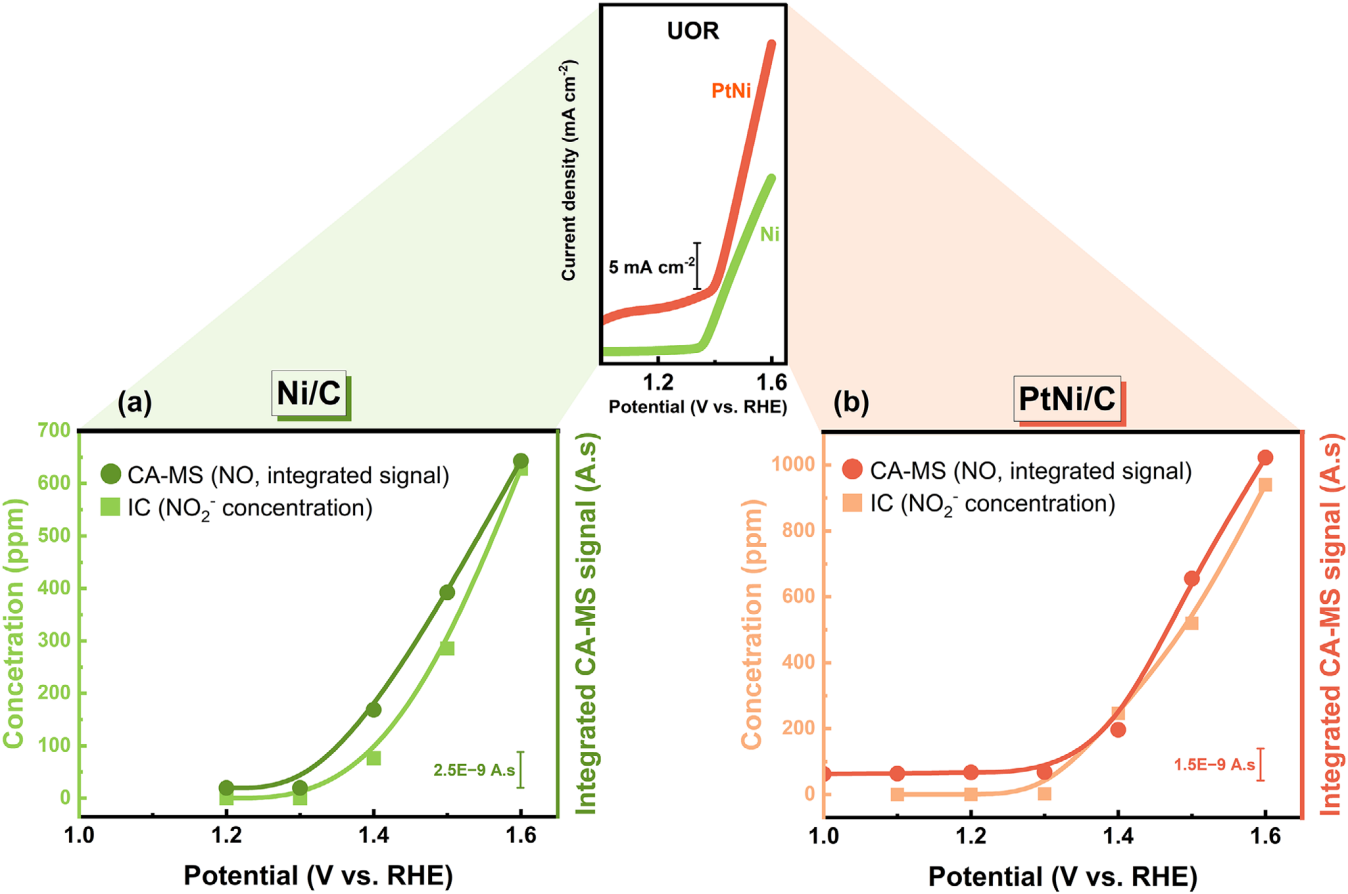
Correlation between electrochemical response and product formation as a function of applied potential. The upper panel shows the current responses of Ni/C (green) and PtNi/C (orange) under controlled potential conditions. Panels (a) and (b) present the comparison between integrated CA‐MS signals (assigned to gaseous NO) and ion chromatography (IC) quantification of NO_2_^−^ for Ni/C and PtNi/C, respectively, as a function of potential.

One notable finding of this study was the strong potential‐dependent correlation among nitrite formation, NO evolution, and current response for Ni/C and PtNi/C catalysts (Figure 5). Although Medvedev et al. proposed that NO_2_^−^ arises from the reaction between NO and hydroxyl species [19], the present findings provided compelling experimental confirmation of this pathway, identifying it as the dominant route under the investigated conditions. These results indicated that nitrite formation predominantly proceeded through NO oxidation (pathway c7). Although the OLEMS and IC experiments were performed in different cell configurations optimized for gaseous and soluble product detection, respectively, the close agreement between the onset potentials of the oxygenated nitrogen species observed by both techniques further supports the reliability of the proposed pathway.

**FIGURE 6:**
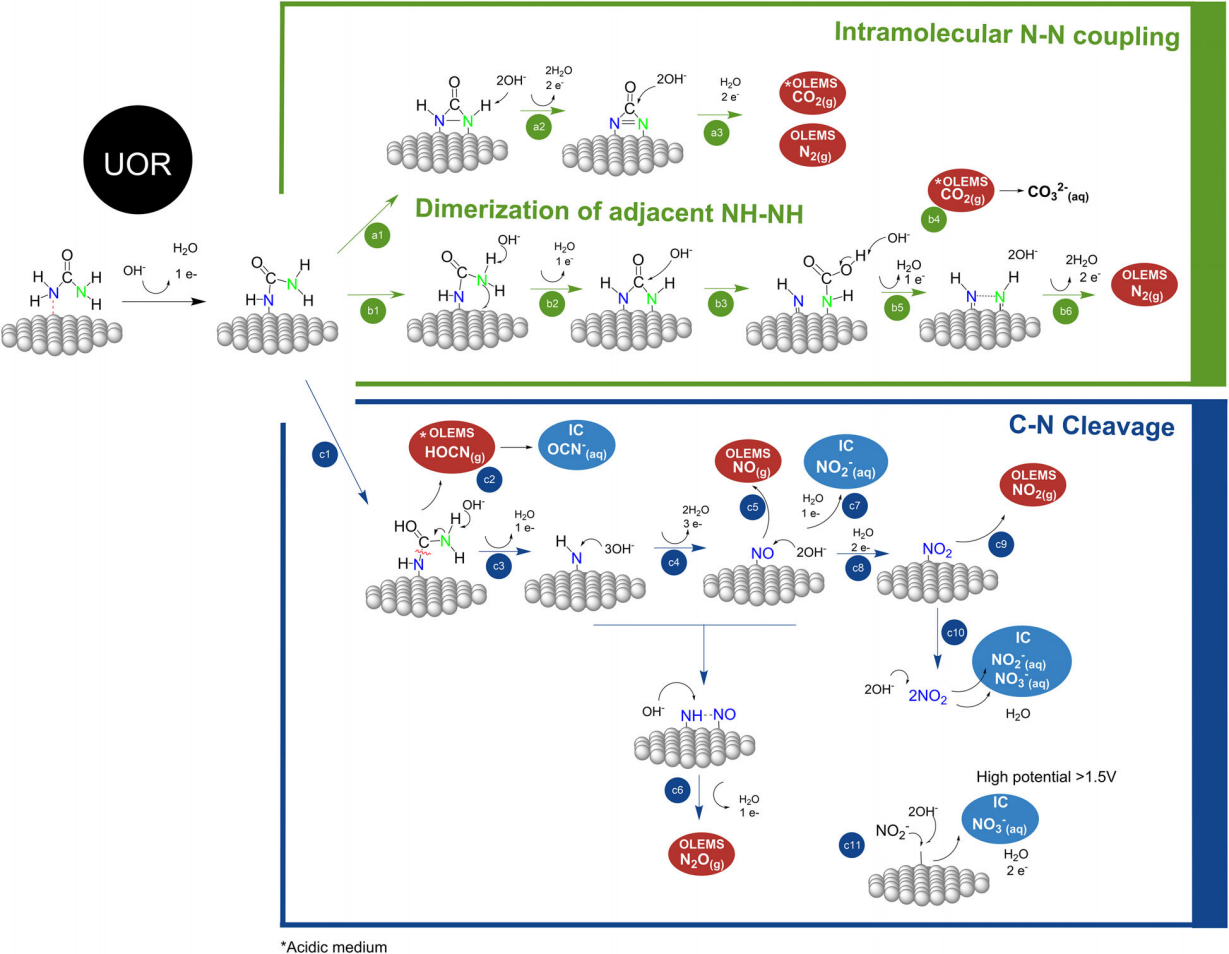
Schematic representation of proposed UOR mechanism pathways on Pt/C, PtNi/C, and Ni/C electrocatalysts.

Thus, based on the experimental evidence obtained, the PtNi/C catalyst stood out for expanding the operational window of the UOR, promoting a potential‐dependent modulation in the distribution of gaseous and soluble products, thereby demonstrating greater catalytic versatility compared with monometallic systems. Accordingly, the bimetallic system integrated the mechanistic characteristics of both metals, making it a promising catalytic platform with potentially superior performance and selectivity for UOR under the investigated conditions.

## 3 Conclusion

This work demonstrated that incorporating Pt into Ni improved the electrocatalytic activity and altered the reaction pathways of urea oxidation compared with monometallic Pt and Ni catalysts supported on carbon. The combination of OLEMS and IC, applied as a function of the potential in both cyclic voltammetry and chronoamperometry, enabled the unequivocal identification of seven gaseous products associated with UOR, together with O_2_ from the competing OER, as well as three soluble species. Six UOR‐derived gaseous products were identified: N_2_, NO, N_2_O, and NO_2_ were detected directly in alkaline media, whereas CO_2_ and HOCN required complementary confirmation under acidic conditions. In parallel, IC enabled the quantification of three solution‐phase species, NO_2_^−^, NO_3_^−^, and OCN^−^, providing a comprehensive assessment of both volatile and dissolved reaction products. The systematic correlation between the identified gaseous and solution‐phase products supported the formulation of an integrated mechanistic framework for UOR, allowing the clear definition of two distinct potential‐dependent reaction mechanisms. In Region I (0.8–1.4 V), platinum played a central role in the adsorption and initial activation of urea, favoring the pathway to N_2_ formation. At higher potentials, corresponding to Region II (1.4–1.6 V), the formation of NiOOH species became dominant, governing the reaction pathway by promoting oxidative steps and favoring the C─N bond cleavage route. This shift directed selectivity toward more oxidized products, both gaseous (NO, N_2_O, and NO_2_) and solution‐phase (NO_2_^−^, NO_3_^−^, and OCN^−^) species, with NO_2_^−^ and OCN^−^ identified as the major products under these conditions. Notably, the results of this study demonstrated a consistent potential‐dependent correlation, supporting NO as a key intermediate in the formation of NO_2_^−^, which emerged as the dominant product at high potentials for Ni/C and PtNi/C. This mechanistic transition highlighted the synergistic effect of the PtNi catalysts, extending the activity window and altering the product distribution across potential regions. These findings demonstrate how catalyst composition directly influences reaction pathways and selectivity, establishing important structure property function relationships for UOR. The use of well‐defined Pt, Ni, and PtNi‐based catalyst systems enabled the identification of intrinsic composition‐dependent effects on activity and product distribution, providing fundamental mechanistic insights into this intrinsically complex reaction. In particular, the results indicate that highly accessible Pt surface sites are essential for promoting UOR at low potentials, thereby modifying the adsorption energetics of key intermediates and contributing to improved N_2_ selectivity. The integration of these mechanistic insights provides valuable guidelines for the rational design of nanostructured anode materials with enhanced low‐potential activity, improved selectivity toward N_2_ formation, and greater operational stability for urea‐assisted water splitting and direct urea fuel cells.

## Conflicts of Interest

The authors declare no conflicts of interest.

## Supporting information




**Supporting File**: smll74274‐sup‐0001‐SuppMat.docx.

## Data Availability

The data that support the findings of this study are available from the corresponding author upon reasonable request.
