# Supplementary material for: Unraveling Potential‐Dependent Mechanistic Pathways of Urea Oxidation on Pt, Ni, and PtNi Electrocatalysts
Source: Small. 2026 Jun 21;22(45):e74274. doi: 10.1002/smll.74274 (PMC13472264; doi:10.1002/smll.74274)
Supplement: Supplementary file 1 [file SMLL-22-e74274-s001.docx]

**Supplementary Information for:**

**Unraveling Potential-Dependent Mechanistic Pathways of Urea Oxidation on Pt, Ni, and PtNi Electrocatalysts**

Rodrigo G. de Araujo,^[a]^ Diego G. Gigliotti,^[a]^ Geraldo N. Tessaro,^[a]^ Seiti I. Venturini,^[a]^ and Joelma Perez*^[a]^

^a^ *University of São Paulo (USP), São Carlos Institute of Chemistry, 13560-970, São Carlos, São Paulo, Brazil*

*Email: [jperez@iqsc.usp.br](mailto:jperez@iqsc.usp.br)

Table of Contents

[1.	Materials and methods	2](#_Toc229736365)

[2.	Physical characterization	3](#_Toc229736366)

[3.	Electrochemical measurements	4](#_Toc229736367)

[4.	Protocols for electrochemical studies	5](#_Toc229736368)

[5.	OLEMS	6](#_Toc229736369)

[6.	IC	6](#_Toc229736370)

[7.	Faradaic efficiency	7](#_Toc229736371)

1. **Materials and methods**

**Chemicals and reagents**

Sodium hydroxide (NaOH, 99.9%, Sigma-Aldrich), Urea (CH_4_N_2_O, 99.5%, Sigma-Aldrich) Isopropyl alcohol (C_3_H_8_O, PA, Honeywell), Nafion solution (C_9_HF_17_O_5_S, 5% (w/w), Aldrich), Potassium bicarbonate (KHCO_3_, 99.7%, Sigma-Aldrich), Sodium carbonate (Na_2_CO_3_, 99.95%, Sigma-Aldrich), Potassium cyanate (KOCN, 96%, Sigma Aldrich), Pt/C 20% (w/w) ETEK, Ni/C 20% (w/w) Fuel cell store, PtNi/C 20% (w/w) Fuel cell store, Sodium nitrate (NaNO_3_, 99.0%, Sigma-Aldrich), Sodium nitrite (NaNO_2_, 99.5%, Sigma-Aldrich), Oxalic acid (C_2_H_2_O_4_, 99.0%, Sigma-Aldrich) and Sulfuric acid (H_2_SO_4_, 96% suprapur^®^, Millipore).

**Characterizations**

The electrocatalysts were characterized using ex-situ techniques, including X-ray diffraction (XRD, Rigaku Ultima IV) to determine the crystallinity and structure of the particles, transmission electron microscopy (TEM, JEM-2100-JEOL) to assess the size and size distribution of the nanoparticles, energy-dispersive X-ray spectroscopy (EDX, LEO-440) to analyze the chemical composition, and thermogravimetric analysis (TGA, METTLER Toledo) to determine the metal loading and thermal stability. The oxidation state of Pt/C, PtNi/C, Ni/C was determined by X-ray photoelectron spectroscopy (XPS) in a Scienta-Omicron ESCA+ spectrometer equipped with a monochromatic Al Kα source (hν = 1486.6 eV), the spectra were analyzed using CasaXPS software (2.3.15) and calibrated to the adventitious carbon peak at 284.8 eV.

**Electrochemical measurements**

Electrochemical characterization was conducted using gas diffusion electrodes (GDEs) made from carbon cloth (geometric area: 1.9 cm^2^). The GDEs were prepared using a metallic load of 1.0 mg_metal_ cm^–2^ dispersed in Isopropyl alcohol and Nafion® (5% w/w) for 20 minutes. The dispersion was uniformly applied to the carbon cloth by drop casting and then dried at 80°C for 1 hour.

For the study of the urea oxidation reaction (UOR), a Teflon cell with a three-electrode configuration was used, in which an Hg│HgO (NaOH, 1.0 mol L^–1^), a graphite rod, and a GDE were used as the reference electrode, counter electrode, and working electrode, respectively. All potentials were converted to the RHE scale. The cell illustration can be consulted elsewhere.^[1]^ The electrolyte used was 1.0 mol L^–1^ NaOH in the presence and absence of 1.0 mol L^–1^ urea. The electrochemical system was purged with argon prior to measurements. The measurement protocols for cyclic voltammetry (CV) and chronoamperometry (CA) are described in Table S1 and S2, respectively.

**Online Electrochemical Mass Spectrometry (OLEMS) measurements**

To detect reaction products during mass spectrometry measurements, the spectrometer capillary was coupled to the electrochemical cell. The spectrometer used was the OmniStar® GSD 301 from Pfeiffer Vacuum, equipped with a Prisma QMS 200, operating with an ionization energy of 70 eV, an emission current of 1 mA, and a pressure on the order of 10^–6^ mbar. The monitored mass signals corresponding to the gaseous products were: *m/z* 28 (N_2_), 30 (NO), 32 (O_2_), 43 (HNCO), 44 (CO_2_ and N_2_O), and 46 (NO_2_). CA-MS and CV-MS were named for the mass signals during electrochemical measurements.

**Ion chromatography analysis**

Electrolysis was performed to evaluate the products of UOR in solution, using Pt/C, Ni/C and PtNi/C, catalysts at the same potential as those used in the OLEMS studies. The experiments were conducted on GDE with an area of 4 cm^2^ in an H-type cell, with compartments separated by a Nafion 115 membrane. Solution samples were collected after 1 hour of electrolysis for subsequent analysis. For each potential, the solution was replaced with a fresh one. The ion chromatography (IC) study was conducted using a Metrohm 930 Compact IC Flex ion chromatograph equipped with a Supp10 column. The mobile phases used were 5.0 mmol L^–1^ KHCO_3_ and 5.0 mmol L^–1^ Na_2_CO_3_ at a flow rate of 1 mL min^–1^, with a suppression solution composed of 200 mmol L^–1^ H_2_SO_4_ and 200 mmol L^–1^ C_2_H_2_O_4_.

In the IC experiments, larger-area electrodes and an H-type cell separated by a Nafion 115 membrane were employed to increase product accumulation and avoid the reduction of anodically formed species at the cathode, thereby enabling more reliable quantification of soluble products. In contrast, the OLEMS configuration was optimized for the real-time detection of gaseous species with high temporal resolution. Therefore, the two techniques were used in a complementary manner, each optimized for a specific type of analysis.

2. **Physical characterization**

Figure S1. Thermogravimetric analysis of Pt, Ni, and PtNi catalysts.

Table S1. EDX and TG analysis for Pt/C, Ni/C, and PtNi/C catalysts.

| Catalyst | EDX | | | TG %_metal_ |
| --- | --- | --- | --- | --- |
|  | %C | %Ni | %Pt |  |
| Pt/C | 80.00 | 0.00 | 20.00 | 21.5 |
| Ni/C | 85.29 | 14.71 | 0.0 | 22.9 |
| PtNi/C | 81.24 | 4.31 | 14.44 | 21.7 |

Table S2. XPS peak position and oxidation state for Pt/C, Ni/C, and PtNi/C catalysts.

| Catalyst | Peak position (eV)  Pt 4f_7/2_ | Oxidation state | Peak position (eV)  Ni 2p_3/2_ | Oxidation state |
| --- | --- | --- | --- | --- |
| Pt/C | 71.2 | Pt^0^ (64%) |  |  |
|  | 72.6 | Pt^2+^ (29%) |  |  |
|  | 74.9 | Pt^4+^ (7%) |  |  |
| Ni/C |  |  | 855.5 | Ni^2+^ (100%) |
| PtNi/C | 71.3 | Pt^0^ (70%) | 853.1 | Ni^0^ (23%) |
|  | 72.7 | Pt^2+^ (26%) | 856.1 | Ni^2+^ (77%) |
|  | 75.0 | Pt^4+^ (4%) |  |  |

3. **Electrochemical measurements**

Figure S2: Cyclic voltammograms of (a) Pt, (b) PtNi, and (c) Ni at 5 mV s^–1^ in 1.0 mol L^–1^ NaOH with and without 1.0 mol L^–1^ urea (insets: magnification of the UOR onset region). (d, e) Comparison of the CVs for all electrocatalysts. Currents were normalized to the geometric surface area.

4. **Protocols for electrochemical studies**

Table S3. Protocols for electrochemical CV-MS studies in the presence and absence of urea.

| Catalyst | Experiment | E_initial_  (V vs. RHE) | E_final_  (V vs. RHE) | Cycles Nº | Scan rate  (mV s^–1^) |
| --- | --- | --- | --- | --- | --- |
| Ni | Cyclic voltammetry | 0.8 | 1.6 | 1 | 1.0 |
| Pt and PtNi | Cyclic voltammetry | 0.1 | 1.6 | 1 | 1.0 |

Table S4. Chronoamperometric (CA-MS) studies with several potentials steps in the presence of urea.

| Experiment | E (V vs. RHE) | | Time (s) |
| --- | --- | --- | --- |
|  | Pt and PtNi | Ni |  |
| Baseline | 0.05 |  | 600 |
| Chronoamperometry | 0.6 |  | 600 |
| Baseline | 0.05 |  | 600 |
| Chronoamperometry | 0.7 |  | 600 |
| Baseline | 0.05 |  | 600 |
| Chronoamperometry | 0.8 |  | 600 |
| Baseline | 0.05 |  | 600 |
| Chronoamperometry | 0.9 |  | 600 |
| Baseline | 0.05 |  | 600 |
| Chronoamperometry | 1.0 |  | 600 |
| Baseline | 0.05 |  | 600 |
| Chronoamperometry | 1.1 |  | 600 |
| Baseline | 0.05 |  | 600 |
| Chronoamperometry | 1.2 | 1.2 | 600 |
| Baseline | 0.05 | 0.0 | 600 |
| Chronoamperometry | 1.3 | 1.3 | 600 |
| Baseline | 0.05 | 0.0 | 600 |
| Chronoamperometry | 1.4 | 1.4 | 600 |
| Baseline | 0.05 | 0.0 | 600 |
| Chronoamperometry | 1.5 | 1.5 | 600 |
| Baseline | 0.05 | 0.0 | 600 |
| Chronoamperometry | 1.6 | 1.6 | 600 |
| Baseline | 0.05 | 0.0 | 600 |

5. **OLEMS**

To confirm the formation of isocyanic acid (HNCO, m/z 43), a complementary CA-MS experiment (Figure S3) was performed in acidic and alkaline medium using a platinum gauze electrode.

Figure S3: CA-MS analyses using a Pt-gauze electrode in 1.0 mol L^–1^ urea solution in two different electrolytes: (a) 0.5 mol L^–1^ H_2_SO_4_ and (b) 1.0 mol L^–1^ NaOH.

6. **IC**

Figure S4: IC chromatograms and calibration curves of the analytical standards. (a) KCNO; (b) NaNO_2_; (c) NaNO_3_.

Figure S5: Quantification of OCN⁻, NO_2_^–^, and NO_3_^–^ in 1.0 mol L^–1^ NaOH + 1.0 mol L^–1^ urea after 1 hour of electrolysis at different applied potentials for: (a) Pt/C, (b) PtNi/C, and (c) Ni/C.

7. **Faradaic efficiency**

The Faradaic efficiency (FE) of products was calculated as follows

$$FE=\frac{n\times F\times c\times V}{Q} \times100\%$$

where *n* is the number of electrons transferred per molecule of product. Specifically, the value of *n* is 6 and 8 for NO_2_^–^ and NO_3_^–^, respectively. *F* is the Faraday constant (C mol^-1^), *c* is the concentration (mol L^-1^), *Q* is the total charge (C), and V volume of the electrolyte (L).

**Reference**

[1] R.G. de Araujo, and J. Perez, “Nitrogen Electrochemical Reduction Reaction Pathways Evidenced by Online Electrochemical Mass Spectrometry and Isotope Labeling on the MoS_2_ Surface,” *ACS Electrochemistry* 1 (2025): 294-302, <https://doi.org/10.1021/acselectrochem.4c00135>.
